# Acute and chronic diabetes complications associated with self-reported oral health: a retrospective cohort study

**DOI:** 10.1186/s12903-020-1054-4

**Published:** 2020-03-07

**Authors:** Kamini Kaura Parbhakar, Laura C. Rosella, Sonica Singhal, Carlos R. Quiñonez

**Affiliations:** 1 Dental Public Health, Faculty of Dentistry, University of Toronto, Toronto, Ontario Canada; 2 Division of Epidemiology, Dalla Lana School of Public Health, University of Toronto, Toronto, Ontario Canada; 3 Institute for Clinical Evaluative Sciences, Toronto, Ontario Canada; 4 Public Health Ontario Toronto, Ontario, Canada

**Keywords:** Oral health, diabetes complications, periodontal disease, Population health, Acute, Chronic

## Abstract

**Background:**

Oral health is associated with diabetes, but the chances of experiencing acute or chronic diabetes complications as per this association is unknown in Canada’s most populous province, Ontario. This study assesses the impact of self-reported oral health on the likelihood of experiencing acute and chronic complications among a cohort of previously diagnosed diabetics.

**Methods:**

A retrospective cohort study was conducted of diabetics (*n* = 5183) who participated in the Canadian Community Health Survey 2003 and 2007–08. Self-reported oral health status was linked to health encounters in electronic medical records until March 31, 2016. Multinomial regression models determined the odds of the first acute or chronic complication after self-report of oral health status.

**Results:**

Thirty-eight percent of diabetics reporting “poor to fair” oral health experienced a diabetes complication, in comparison to 34% of those reporting “good to excellent” oral health. The odds of an acute or chronic complication among participants reporting “poor to fair” oral health status was 10% (OR 1.10; 95% CI 0.81, 1.51) and 34% (OR 1.34; 95% CI 1.11, 1.61) greater respectively, than among participants experiencing no complications and reporting “good to excellent” oral health.

**Conclusion:**

Self-reporting “poor to fair” oral health status is associated with a greater likelihood of chronic complications than acute complications. Further research regarding the underlying causal mechanisms linking oral health and diabetes complications is needed.

## Background

Diabetes is a major public health concern in Canada and worldwide. Characterized by hyperglycemia and the insufficient secretion or action of insulin, diabetes is a metabolic disorder that amplifies inflammatory immune responses resulting in diabetes complications [1, 2]. The Canadian prevalence of diabetes currently sits at 3.65 million and is predicted to increase by over 30% within the next decade, and this is coupled with growing health care costs, morbidity, and premature mortality [3]. In Ontario, Canada’s most populous province, the current prevalence of diabetes surpasses national estimates, arguably necessitating public health action [4].

Since the U.S. Surgeon General’s Report on oral health in 2000, which placed a heavy emphasis on the relationship between oral health and systemic health, the link between diabetes and periodontal disease has received considerable attention [5]. Periodontal disease is a chronic inflammatory condition characterized by the destruction of oral tissues supporting teeth [6]. Although periodontal disease is most commonly known as a complication of diabetes, the evidence indicates that the link between these two conditions may be bidirectional [7, 8]. Initiated by microbial dysbiosis, periodontal disease produces a low grade host immune response that can stimulate systemic inflammation [9, 10]. Specifically, the shift in symbiotic microbial communities of the periodontal tissues accounts for an exaggerated immune defense mechanism leading to the destruction of periodontal tissues [9, 10]. In turn, among diabetics, hyperglycemia accounts for an alteration in cell function, defective neutrophil apoptosis, oxidative stress, and the excessive production of inflammatory mediators that exacerbate insulin resistance resulting in health complications [6, 11, 12]. A unified inflammatory mechanism between periodontal disease and diabetics is thought to impact diabetes health outcomes and complications [13, 14].

Diabetes complications are classified as acute and chronic, and chronic complications are further classified as microvascular or macrovascular in nature [13]. Acute complications of diabetes include hyperglycemia and hypoglycemia, and chronic complications include cardiovascular diseases, kidney failure, retinopathy and neuropathy [13]. Diabetes complications with the highest prevalence and greatest cost to the Canadian health care system are predominantly chronic and include stroke, myocardial infarction, kidney failure, lower limb amputations and vision loss [3, 15]. Although the biological mechanism of acute and chronic complications has been long debated, there is some consensus on the differences between them and the possibility of a continuum from acute to chronic [13, 16]. Acute complications of diabetes have been characterized by changes in metabolic control, specifically hyperglycemia [13, 17]. In contrast, chronic hyperglycemia is commonly assumed to be the central determinant in chronic diabetes complications. However, insulin resistance and macrovascular damage are found to play a large role [13, 17].

In the mechanism linking periodontal disease to diabetes, the role of insulin resistance and exaggerated immune inflammatory pathways have been considered the driving force in the “bidirectional” link [1, 2, 18–20]. To quantify the periodontal impact on diabetic health, studies have explored the effect that periodontal treatment has on blood sugar levels and lipid profile markers [2, 19, 21, 22]. The current evidence for periodontal treatment among diabetics also suggests that periodontal disease may play the role of a risk factor, as periodontal treatment was found to reduce blood sugar levels as well as cholesterol levels and high-density lipids [21]. Periodontal care has also been associated with reductions in health complications, hospital admissions and overall medical and pharmaceutical costs among diabetics [2, 23–25]. However, there is no research using Canadian populations in relation to oral health and diabetes, and there is a paucity of population level evidence supporting the epidemiological association between oral and diabetes health generally [26]. This study thus aims to identify the odds of future acute and chronic complications among diabetics who have self-reported oral health status in Canada’s most populous province, Ontario.

## Methods

### Study design

A retrospective cohort study was designed to explore the odds of acute and chronic diabetes complications among Ontario residents self-reporting oral health status. Study participants were selected from a combined pool of respondents interviewed from the 2003 and 2007–08 Canadian Community Health Survey (CCHS) [27]. Briefly, the CCHS is a national survey that utilizes a multi-stage, stratified, clustered-probability survey sampling design and is representative of 98% of the Canadian population [28]. The survey is administered by Statistics Canada and collects self-reported health information from Canadians over the age of 12, excluding those living on indigenous reserves, residing in institutions and full-time members of the Canadian armed forces [28]. Details regarding the CCHS methodology are documented elsewhere [28].

The study cohort was restricted to Ontario residents over the age of 40 with a validated diabetes diagnosis. As all residents of the province of Ontario are covered by a single payer insurance system, the Ontario Health Insurance Plan (OHIP), health system encounters among these participants can be followed. With de-identified health card numbers, health encounters in electronic medical records held by the Institute for Clinical Evaluative Sciences (ICES) were followed from the initial CCHS interview date, until March 31, 2016. A validated diabetes diagnosis was confirmed using the Ontario Diabetes Database (ODD), an ICES-derived disease registry containing physician diagnosed cases of diabetes in Ontario [29].

The final study sample was composed of individuals over the age of 40, who participated in the oral health component of the CCHS and had an ODD confirmed diagnosis of diabetes. Individuals were excluded if they could not be linked to electronic medical records, were OHIP-ineligible during the follow-up period, or if they did not report oral health status during survey administration. The final analytical sample consisted of 5183 participants, which represents a weighted sample of 1.31 million Ontario residents. Ethical approval for this study was obtained from the University of Toronto Research Ethics Board and was followed by ICES approval for data creation and access at Sunnybrook Hospital in Toronto, Ontario (protocol reference no. 34553).

### Oral health status

CCHS cycles 2003 and 2007–08 were selected for the availability of oral health data for the province of Ontario [28]. Self-reported oral health status was assessed through the question “would you say the health of your teeth and mouth is: excellent, very good, good, fair or poor?”. According to the distribution of self-reported oral health status among study participants and to determine the differences among positively and negatively inclined oral health responses, the exposure variable was re-categorized into two values representing “good to excellent” and “poor to fair” oral health categories. Further description of oral health content from the CCHS is described elsewhere [28].

### Diabetes complications

The primary outcome of this study was the first diabetes complication experienced by participants after the CCHS interview date. Complications were extracted from hospitalization and emergency department records. International Classification of Disease (ICD-9) codes, were used to extract diabetes-specific complications from these data [30]. Acute complications included non-specific hypoglycemia and hyperglycemia; chronic complications included myocardial infarction, stroke, skin infections, amputation, dialysis, and retinopathy. Following the CCHS interview, participants were classified in three categories comprising of those who did not experience any complication, those who experienced an acute complication and those who experienced a chronic complication.

### Covariates

CCHS participants’ demographic characteristics, health behaviours and medical histories, were included in the study as covariates, according to their association with oral health and diabetes outcomes. Covariates included the following: age, sex, income, education, rural/urban index, race, physical activity, smoking status, alcohol consumption, dental visits, basal metabolic index (BMI), prior co-morbidity, duration of diabetes prior to interview date, stress, community sense of belonging, health care visits prior to the complication and self-reported overall health.

Age, BMI and duration of diabetes were continuous measures while all other covariates were categorical. The duration of diabetes prior to interview date was used to consider the impact of a dated or early diabetes diagnosis. The Rurality Index of Ontario (RIO) was used to classify individuals residing in rural or urban areas. Participants with RIO scores < 39 were categorized as urban residents and scores ≥40 were categorized as rural residents [31]. Co-morbidity at the interview date was assessed from CCHS questions regarding chronic diseases and participants were classified into two categories comprising of participants without any co-morbidity and participants with any of the following conditions: arthritis, chronic obstructive pulmonary disease (COPD), heart disease and stroke. Health care visits was assessed by extracting OHIP codes for visits to a general practitioner or specialist. Visits were classified into three categories, comprising of participants who visited their general practitioner, specialist or both general and specialist practitioners for diabetes management.

### Statistical analysis

Starting with baseline characteristics, all variables were assessed for their association with self-reported oral health. T-tests were used to express the means and standard deviations of continuous variables and Chi-squared tests were used to express the cross tabulated frequencies of categorical variables. Bivariate analysis was conducted by the multi-categorical outcome. Furthermore, variables with a *p*-value ≤0.25 following the baseline and bivariate analysis were included and the most parsimonious model was built [32]. Variables that did not reach a p-value of ≤0.25 but were associated with the exposure or outcome in the literature were also included in the model. Multinomial logistic regression models were used to determine the odds of experiencing an acute or chronic outcome following the interview date among participants reporting “poor to fair” oral health. Multinomial odds ratios with confidence intervals were estimated for diabetes complications associated with oral health, where individuals reporting “good to excellent” oral health status, whom did not experience any diabetes complications following the interview date, represented the reference group. Oral health was the explanatory variable and covariates were included in the model if they were clinically significant or were associated with diabetes outcomes in existing literature.

Bootstrapping sample weights provided by Statistics Canada were applied to the analysis to adjust for the complex nature of the CCHS survey design. This generated inferable estimates for the Ontario population. All statistical analyses were performed using SAS version 9.4 and data was accessed at Sunnybrook Hospital ICES Central in Toronto, Canada. PROC SURVEY MEANS, PROC SURVEYFREQ and PROC SURVEYLOGISTIC were used in the analysis [33].

## Results

A total of 5183 diabetics over the age of 40 were followed through electronic medical records at ICES. As shown in Table 1, 38% of those reporting “poor to fair” oral health experienced a diabetes complication. The prevalence of chronic complications in this subgroup was 33%. In contrast, 34% of those reporting “good to excellent” oral health experienced a complication. The prevalence of chronic complications in this subgroup was approximately 29%. Both groups experienced a similar frequency of acute complications at approximately 5%.

**Table 1:** Type of diabetes complications experienced by participants self-reporting their oral health status (*n* = 5183; *N* = 1,308,911)

Oral Health Status	Complication Type			Total
	No complication	Acute complication	Chronic complication	
Good to Excellent	2693 (66%)	199 (5%)	1198 (29%)	4090
Poor to Fair	674 (62%)	55 (5%)	364 (33%)	1093
Total	3367	254	1562	5183

Baseline characteristics of participants according to the diabetes outcome type are shown in Table 2. Participants who did not experience a complication were a mean age of approximately 62 years, while those who experienced an acute or chronic complication were a mean age of approximately 65 years. A majority of those who did not experience a complication were also male and self-reported higher income. The study sample was predominantly composed of individuals self-identifying as white, living in urban areas and having a post-secondary education. A greater majority of those who experienced an acute or chronic complication reported having other chronic diseases as well as a higher BMI and a longer duration of diabetes prior to the interview date. They were also current smokers, consumed alcohol regularly, lived predominantly inactive lifestyles and had not seen a dentist in the past 12 months.

**Table 2:** Baseline weighted characteristics of study participants according to type of complication outcome (*n* = 5183; *N* = 1,308,911)

Baseline Characteristics	Diabetes Complications		
	No Complication (***n*** = 3367)	Acute Complication (***n*** = 254)	Chronic Complication (***n*** = 1562)
**Age** (Mean, ±SD)	61.6 ± 0.4	64.8 ± 1.4	65.6 ± 0.7
**Sex** (% Male)	51.8	42.2	44.1
**Race** (%White)	72.7	80.9	82.4
**Income** (%)			
Quintile 1	14.7	19.0	18.1
Quintile 2	13.2	9.8	15.1
Quintile 3	20.4	16.2	17.8
Quintile 4	19.2	17.7	22.0
Quintile 5	17.9	21.1	14.7
**Education** (%)			
< Diploma	21.0	33.8	31.9
Diploma	12.3	13.1	16.3
Post-Secondary	61.6	48.4	49.2
**RIO** (%)			
Urban	92.3	91.0	87.7
Rural	7.7	9.0	12.4
**Chronic disease** (%)	49.6	65.8	64.4
**BMI** (Mean, SD)	27.8 ± 0.2	28.4 ± 0.8	28.6 ± 0.3
**DM duration** (years) (Mean, SD)	6.4 ± 0.2	10.4 ± 0.5	7.4 ± 0.2
**Stress** (%)	43.2	38.7	39.9
**Health status** (%)			
Excellent	6.5	3.9	4.7
Very good	21.9	8.1	16.1
Good	38.2	33.1	31.0
Fair	23.4	32.6	30.8
Poor	10.0	22.3	17.9
**Community belonging** (%)	66.7	65.5	64.4
**Smoking** (%)			
Current	13.5	21.3	17.6
Former	46.8	46.8	48.2
Never smoked	39.7	33.3	34.2
**Alcohol use** (%)			
Regular	46.2	31.9	41.2
Occasionally	18.3	23.8	21.7
Former	30.8	39.8	31.4
Never drank	4.7	4.5	5.9
**Activity index** (%)			
Active	17.9	14.4	17.7
Mod. active	21.9	13.7	19.1
Inactive	60.2	71.8	63.1
**Dental visits** (%)			
0/year	37.6	46.2	45.8
1–2 Visits/year	45.5	42.2	40.1
> 2 Visits/year	16.9	11.7	14.1
**Health care visits** (%)			
General Physician	11.1	2.3	3.1
Specialist	8.7	15.6	12.2
GP + SP	80.2	82.1	84.7

*Note: percentages may not add up to 100% because of missing categories or rounding

The odds ratios shown in Table 3 depict the difference in the likelihood of an acute or chronic diabetes complication among study participants. In the fully adjusted multinomial regression, the odds of an acute complication versus no complication among participants reporting “poor to fair” versus “good to excellent” oral health was 10% greater [OR = 1.10, 95% CI 0.81, 1.51]. The odds of those experiencing a chronic complication in the final model, versus no complication among participants reporting “poor to fair” versus “good to excellent” oral health was 34% greater [OR = 1.34, 95% CI 1.11, 1.61].

**Table 3:** Odds ratios and 95% confidence intervals from a fully adjusted multinomial logistic regression for the relation between self-reported oral health and diabetes complications

	Diabetes Complications			
	Acute vs. No complication		Chronic vs. No complication	
	Odds Ratio	95% CI	Odds Ratio	95% CI
**Self-reported oral health**				
Good to excellent	1.00	–	1.00	–
Poor to fair	1.10	0.81, 1.51	1.34	1.11, 1.61
**Age**	1.00	0.99, 1.02	1.02	1.01, 1.03
Age*Sex				
Male	1.00	–	1.00	–
Female	1.00	1.00, 1.01	0.99	0.99, 0.99
**Race**				
White	1.00	–	1.00	–
Ethnic minority	1.12	0.73, 1.70	0.76	0.58, 0.99
**Income**				
Quintile 1	0.34	0.21, 0.55	0.94	0.71, 1.25
Quintile 2	0.30	0.19, 0.49	1.08	0.80, 1.46
Quintile 3	0.35	0.22, 0.54	0.69	0.54, 0.89
Quintile 4	0.46	0.30, 0.71	1.04	0.81, 1.33
Quintile 5	1.00	–	1.00	–
**Education**				
< Diploma	2.39	1.85, 3.10	1.61	1.28, 2.03
Diploma	1.53	1.04, 2.24	1.69	1.37, 2.10
Post-secondary	1.00	–	1.00	–
**Rurality index of Ontario**				
Urban	1.00	–	1.00	–
Rural	1.18	0.84, 1.65	1.48	1.26, 1.74
**Chronic disease**	1.05	0.81, 1.36	1.35	1.15, 1.59
**BMI**	1.00	0.98, 1.02	1.01	1.00, 1.02
**DM duration**	1.22	1.18, 1.25	1.03	1.01, 1.04
**Self-reported general health**				
Excellent	1.00	–	1.00	–
Very good	0.44	0.04, 5.19	0.83	0.57, 1.20
Good	1.06	0.09, 12.64	0.87	0.60, 1.26
Fair	1.32	0.11, 15.78	1.11	0.78, 1.59
Poor	1.76	0.14, 22.56	1.53	1.01, 2.33
**Smoking**				
Current	1.77	1.08, 2.88	1.17	0.90, 1.51
Former	1.06	0.77, 1.46	0.90	0.76, 1.07
Never smoked	1.00	–	1.00	–
**Dental Visits**				
0/year	1.68	1.19, 2.39	1.27	1.04, 1.55
1–2 Visits/year	1.68	1.62, 2.44	1.05	0.84, 1.31
> 2 Visits/year	1.00	–	1.00	–
**Alcohol use**				
Regular	1.05	0.40, 2.75	0.63	0.40, 1.00
Occasionally	1.82	0.69, 4.75	0.86	0.53, 1.41
Former	1.43	0.57, 3.59	0.64	0.41, 1.01
Never drank	1.00	–	1.00	–
**Health care visit**				
General Physician	0.73	0.18, 2.94	0.38	0.20, 0.70
Specialist	1.52	0.67, 3.45	1.36	0.99, 1.87
GP + SP	1.00	–	1.00	–

Among covariates included in the multinomial model, age and income differences among participants were not significantly associated with the likelihood of acute and chronic complications. As sex was not found to be associated with the study outcome in the bivariate analysis, it was included in the fully adjusted model as an interaction term. However, with every unit increase in age among males and females, there was no effect on the likelihood of acute or chronic complications versus no complications. Trends are observed among education levels, self-reported general health, smoking and dental visits. For education levels, in comparison to those with a post-secondary education, those reporting having a secondary school diploma or less, had a higher likelihood of acute and chronic complications. Similarly, the further away from “good to excellent” a study participant self-reported their general health, the greater their odds for complications. Fewer dental visits and current smoking showed similar trends in comparison to those who visited the dentist more than twice in the past year and those who never smoked, respectively. A trend among those consuming alcohol regularly and occasionally was associated with greater odds of acute complications only.

Individuals identifying as an ethnic minority had higher odds for acute complications and lower odds for chronic complications. Interestingly, those who were living in rural areas, in comparison to those in urban areas, had a higher likelihood for chronic than acute complications. For every unit increase in BMI, there was only a slight but insignificant increase in the odds for chronic complications in comparison to acute complications. Those who reported having comorbidities prior to the interview date showed a similar trend leaning towards greater odds of chronic complications. However, those who had diabetes for a longer duration prior to the interview date were observed to have higher odds for acute complications than chronic complications. Those who were found to have had contact with only a general physician prior to any complication were at a lower likelihood of experiencing acute or chronic complications. In contrast, those who only had contact with a specialist were at a higher risk. Those who had contact with both a general physician and specialist were considered the reference group.

## Discussion

This study’s findings indicate that “poor to fair” self-reported oral health is associated with a greater likelihood for chronic complications than acute complications, after adjusting for an extensive range of covariates. This is in line with studies that have found that diabetics with periodontal disease or those who do not receive periodontal treatment, incur higher medical costs and a greater number of hospitalizations, and supports the association between periodontal disease and chronic diabetes complications [24, 25, 34–37]. This study provides insights about the likelihood for complications among diabetics in Ontario, Canada.

Over the last century, many hypotheses have been developed to explain the oral-systemic link, based on the microbial dysbiosis of periodontal disease [38]. Oral pathogens are thought to impact systemic health by either direct invasion or the indirect stimulation of immune-inflammatory responses [38, 39]. The inconsistency of evidence on direct invasion lead scientists to find more support for the hypothesis of indirect invasion, which may explain the bidirectional impact of periodontal disease [36, 38, 39]. Although our study does not seek to support one hypothesis over the other, nor does it claim causation, the greater likelihood of chronic complications in this study may be explained by the proposition that indirectly exaggerated immune inflammatory responses link periodontal disease to diabetes [40–42].

However, in order to interpret the greater odds of chronic complications than acute complications observed among diabetics reporting “poor to fair” oral health status, it is important to understand the difference between the mechanism of acute and chronic diabetes complications. The literature suggests that the basis of acute diabetes complications is metabolic imbalances and hyperglycemia [13, 17]. Chronic complications are further characterized by insulin resistance that can lead to micro- and macrovascular damage [13, 16, 17, 43]. Although clinical trials have explored the impact of periodontal treatment on the reduction of blood sugar levels, only a few have explored the reduction of lipid markers such as cholesterol, triglycerides and high-density lipids, which are important contributors of insulin resistance and are associated with greater chronic complications [44–48]. Some studies also support the use of BMI as a predictive measure for insulin resistance, however, BMI was not found to have a significant effect on either acute or chronic complications in this current study [49]. As insulin resistance may be the connecting factor for periodontal disease and chronic diabetes complications, further research into this component of the putative bidirectional link may provide insight into preventive measures. At the public health level, for instance, where health outcomes, health expenditures and improved quality of life is of concern, this may provide support for concurrent demands for improvements in access to dental care in Canada and better management of diabetes, reductions in inefficient healthcare costs, such as physician and emergency department use for oral health-related complaints [50, 51], and improvements to the quality of life of diabetics [52].

This study presents with several strengths. Primarily, it has been conducted at the population level, allowing for population-based inferences for diabetics over the age of 40. Second is the use of a validated diabetes diagnosis, which contrasts most of the current literature [24, 25]. In comparison with observational studies, our retrospective selection of participants and longitudinal follow-up allows for arguments beyond a simple association between self-reported oral health and diabetes complications. The use of self-reported oral health also presents as a strength in this study. As self-reported oral health is multi-faceted in nature, representing the social, psychosocial, economic and cultural components of oral health, it presents as a convenient measure for exploring the diabetes health experience [53–55]. Notably, studies have found that self-reported oral health status is consistent with the clinical need for oral treatment [56]. Among diabetics, this measure can thus arguably be used to assess the odds of diabetes complications and provide a means to improve health literacy and expand the referral network for diabetics in need of dental care [57].

Despite these strengths, there are also important limitations to consider. Although self-reported oral health status can be an effective measure to predict oral health needs, it may not be a competent measure for clinical periodontitis [54]. The evidence shows that self-reported oral health can be highly specific but not sensitive [56]. Thus, the current study’s participants may be able to report that they do not have periodontal disease with greater accuracy that those with the condition [56]. However, the evidence also suggests that diabetics may be more aware of their periodontal disease status [58, 59]. Although clinical measures of periodontal disease and diabetes control would provide greater power to our study, such measures have not been linked to electronic medical records in Canada. Using the ODD also presents with the limitation that this database does not differentiate between type 1 or 2 diabetics [29]. As the periodontal disease-diabetes link is based on the pathological mechanism of adult onset diabetes, this limitation may overestimate results; however, as more than 95% of the ODD is made up of type 2 diabetics and the sample was restricted to participants over the age of 40, the impact of type 1 diabetics is minimal and does not arguably impact the study results.

## Conclusion

Overall, this study’s findings support the need for understanding the relationship between oral health status and diabetes complications. Within its limits, “poor to fair” oral health was found to be associated with a greater likelihood of chronic complications than acute complications, providing an important insight for diabetes health in Ontario, Canada.

## Data Availability

The linked data used for this study is held securely in coded form at the Institute for Clinical Evaluative Sciences (ICES). ICES is a not-for-profit research institute whose mandate is to enable health system evaluation and research. ICES has custody and control of a array of Ontario health information (“ICES data”), collected and used by ICES in accordance with applicable law, research ethics approvals and contractual commitments. As a prescribed entity under s. 18(1) of O.Reg 329/04 of Ontario’s Personal Health Information Protection Act (PHIPA), ICES is authorized to disclose ICES data for research that is described in a research plan that meets the requirements of s. 44(2) of the Act and approved by a research ethics board, or in the case of research approved outside of Ontario, for research that meets the requirements of s. 44(10). All administrative data received and held at ICES are used only for the approved research. Only de-identified variables, specific to answer the study questions, are used for the above purpose. Variables are pre-defined in a standardized Dataset Creation Plan (DCP). ICES Data are held on secure ICES servers, located in a restricted area within ICES’ locked and 24/7/365 video-monitored facility. Authorized researchers access ICES data remotely through a secure, encrypted VMware Virtual Desktop, requiring two-factor authentication. ICES data cannot be copied or transferred from the VMware Virtual Desktop, except for results of analysis of ICES data, which may be removed subject to ICES’ approval. Furthermore, no data will be released which could potentially be used in the reidentification of individuals. For example, small cells (consisting of between one and five people) must be suppressed. These ICES methodologies are in place to minimize the risk of identity and attribute disclosure. To request data, please contact Melissa Chiarelli; Research Program Coordinator, Primary Care & Population Health; melissa.chiarelli@ices.on.ca; 416–480-4055 × 6047.

## Abbreviations

BMI: Basal metabolic index
CCHS: Canadian Community Health Survey
COPD: Chronic obstructive pulmonary disease
ICD: International Classification of Disease
ICES: Institute for Clinical Evaluative Sciences
ODD: Ontario Diabetes Database
OHIP: Ontario Health Insurance Plan
RIO: Rurality Index of Ontario
