# Correction to: Acute and chronic diabetes complications associated with self-reported oral health: a retrospective cohort study

**DOI:** 10.1186/s12903-021-01535-x

**Published:** 2021-04-12

**Authors:** Kamini Kaura Parbhakar, Laura C. Rosella, Sonica Singhal, Carlos R. Quiñonez

**Affiliations:** 1 Dental Public Health, Faculty of Dentistry, University of Toronto, Toronto, Ontario Canada; 2 Division of Epidemiology, Dalla Lana School of Public Health, University of Toronto, Toronto, Ontario Canada; 3 Institute for Clinical Evaluative Sciences, Toronto, Ontario Canada; 4 Public Health Ontario Toronto, Ontario, Canada

## Correction to BMC Oral Health (2020) 20:66 https://doi.org/10.1186/s12903-020-1054-4

Following publication of the original article [1], the authors identified an error in the Competing interests section: “Carlos Quiñonez receives consulting income for dental care related issues from Green Shield Canada” should be added.

The original article has been updated.

## References

[1] Parbhakar KK, Rosella LC, Singhal S, Quiñonez CR (2020). Acute and chronic diabetes complications associated with self-reported oral health: a retrospective cohort study. BMC Oral Health.

